# RSV infection-associated pediatric generalized pustular psoriasis with innate immune activation: a case report

**DOI:** 10.3389/fimmu.2026.1808692

**Published:** 2026-04-17

**Authors:** Xiaoyu Zhou, Ran An, Pengting Qu, Qianwen Wang, Wenkang Cheng, Siyan Liu, Junhong Zhao, Haixia Jing

**Affiliations:** 1 Department of Dermatology, Taihe Hospital, Hubei University of Medicine, Shiyan, Hubei, China; 2 Taihe Hospital, Hubei University of Medicine, Shiyan, Hubei, China; 3 School of Pharmacy, Guizhou University of Traditional Chinese Medicine, Guiyang, Guizhou, China; 4 First Clinical College, Hubei University of Medicine, Shiyan, China

**Keywords:** generalized pustular psoriasis, RSV, infant, IL-36, cytokine storm, viral trigger, innate immunity

## Abstract

Generalized pustular psoriasis (GPP) in infancy is uncommon and is often precipitated by infection, drugs, or systemic inflammatory responses. Although viral illnesses such as influenza have been reported to trigger GPP, acute exacerbation secondary to respiratory syncytial virus (RSV) is rarely described. We report a case of a 2-year-old girl with a prior history of mild psoriasis who developed widespread sterile, small pustules several days after onset of RSV infection symptoms (cough and low-grade fever), consistent with an RSV-associated acute exacerbation of GPP. Laboratory testing was positive for RSV nucleic acid and showed markedly elevated inflammatory markers. The family denied any use of over-the-counter medications prior to rash onset; based on the timing of lesion appearance and characteristic histopathology on skin biopsy (subcorneal pustules, Kogoj pustules, and psoriasiform epidermal hyperplasia), acute generalized exanthematous pustulosis (AGEP) was excluded. Treatment and follow-up: the patient received oral acitretin combined with intravenous immunoglobulin and albumin, after which the cutaneous lesions resolved rapidly. At 1-month post-discharge follow-up, the condition remained stable without recurrence. RSV infection should be recognized as a potential trigger for acute exacerbation of GPP in young children. In febrile acute pustular eruptions, viral nucleic acid testing and histopathological examination are important to enable early, targeted intervention.

## Introduction

Generalized pustular psoriasis (GPP) is an acute, relapsing disorder characterized by widespread sterile pustulation and marked systemic inflammation and is clinically regarded as a distinct subtype rather than a mere variant of plaque psoriasis (1). Pediatric GPP is relatively uncommon and is frequently precipitated by infection, abrupt withdrawal of medications, vaccination, or systemic stressors (2). Although viral triggers such as influenza virus and adenovirus have been reported, GPP outbreaks secondary to respiratory syncytial virus (RSV) are rarely described (3). RSV is capable of eliciting robust innate immune responses, including activation of IL-1 family cytokines. However, IL-36 signaling is a well-established central pathway in GPP pathogenesis, and viral infection may act to amplify rather than initiate this axis (4). In infants with potential susceptibility of the IL-36 pathway, such infection may function as the final precipitating event that triggers a GPP flare (“second hit”) (5). We therefore report a case of RSV-associated exacerbation of GPP in a 2-year-old child, with the aim of increasing clinical awareness of virus-triggered pustular eruptions.

## Case presentation

A 2-year-old girl with a history of mild psoriasis was admitted. There was no family history of psoriasis and no recent history of vaccination. Due to lack of regular medical management, no standardized treatment was administered, and only intermittent application of topical corticosteroid ointment was used. There was no history of systemic corticosteroid use or withdrawal. Two weeks prior to admission, she developed cough accompanied by low-grade fever, followed by the appearance of small pustules along the margins of pre-existing psoriatic lesions. The caregivers explicitly denied the use of any over-the-counter cold remedies, antipyretics, or traditional herbal medicines prior to the onset of the rash. The patient had received empirical antibacterial therapy with ceftriaxone (dose unknown) at a local clinic; however, the skin lesions did not improve and instead progressed rapidly.

**Figure 1:**
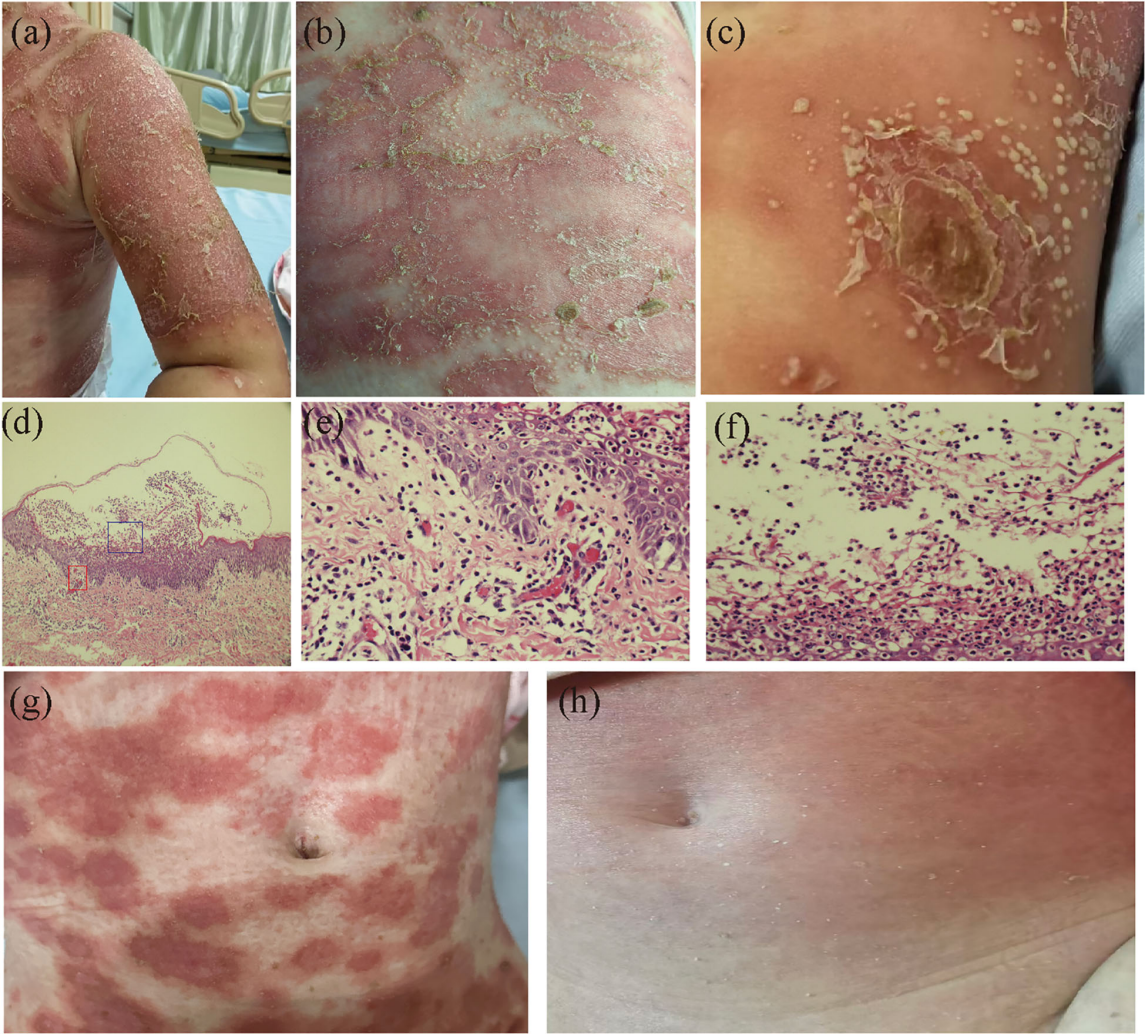
Clinical and histopathological findings of the patient. **(A-C)** Clinical presentation at admission: diffuse erythema over the trunk and extremities, accompanied by numerous superficial sterile pustules with coalescence into “lakes of pus”. **(D-F)** Histopathological findings (hematoxylin and eosin staining). **(D)** Low-power view (×100) of a pustular lesion showing subcorneal pustules, typical spongiform pustules of Kogoj, psoriasiform epidermal hyperplasia, and papillary dermal edema. **(E)** High-power view (×400) of the superficial dermis (red box in d), showing a predominantly neutrophilic infiltrate with only occasional eosinophils scattered in the superficial dermis. **(F)** High-power view (×400) of the intraepidermal pustule (blue box in d), showing a dense neutrophilic collection with no evident eosinophilic infiltration. **(G)** After 13 days of treatment: near-complete resolution of cutaneous lesions with marked fading of erythema. **(H)** Follow-up image: complete clearance of lesions 1 month after discharge, with only mild residual pigmentation and erythema.

On admission, the patient had persistent low-grade fever, with a maximum temperature of 37.7°C lasting for five days, consistent with systemic inflammation in GPP. The patient also exhibited mild muscle weakness, slight pruritus, increased skin temperature, signs suggestive of a burning sensation of the skin, and tachycardia, as assessed based on caregiver observations and clinical evaluation given the patient’s young age. Physical examination revealed widespread erythema over the trunk and extremities, covered with numerous superficial sterile pustules ranging from pinhead to millet size, some of which coalesced to form so-called “lakes of pus” (**Figures 1A–C**). Chest computed tomography demonstrated bronchopneumonia. Laboratory investigations showed a white blood cell count of 15.01 × 10^9^/L (reference range: 3.5–9.5 × 10^9^/L), neutrophils 12.32 × 10^9^/L (reference range: 1.8–7.5 × 10^9^/L), and a C-reactive protein level of 93.69 mg/L (reference range: <5 mg/L). RSV PCR testing was performed using a nasopharyngeal swab specimen and yielded a positive result, with a cycle threshold (Ct) value of 31. Blood cultures and cultures of pustular contents yielded no bacterial growth.

**Figure 2:**
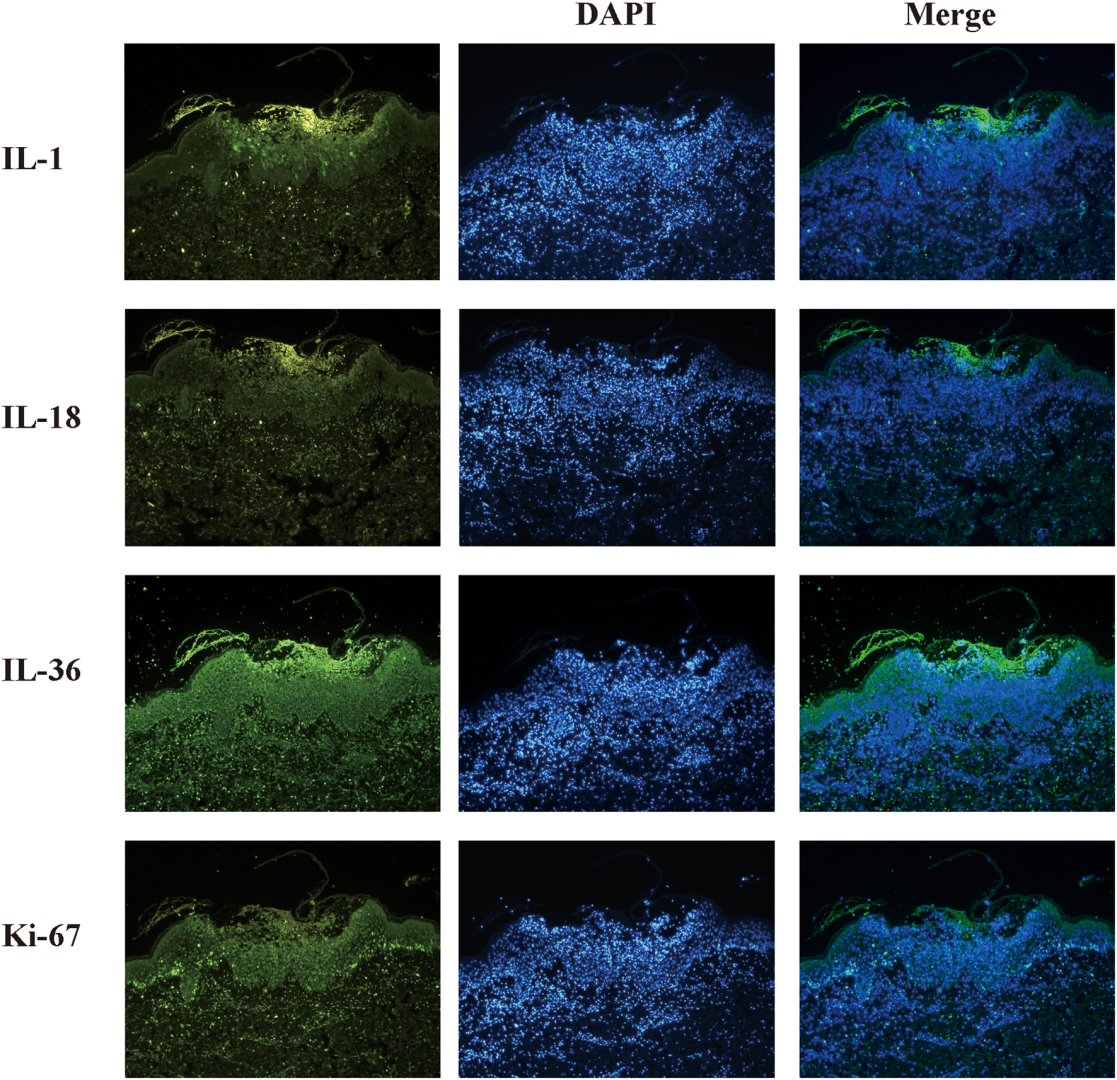
Immunofluorescence analysis of IL-18 and IL-1 in lesional skin. Immunofluorescence staining of lesional skin for IL-1 family cytokines and proliferation marker. Rows show representative fields for IL-1, IL-18, IL-36, and Ki-67. For each marker the left column is the marker signal (green), the middle column is DAPI nuclear counterstain (blue), and the right column is the merged image. IL-36 expression is markedly increased in the epidermis, particularly within pustular regions, and is also observed in the dermis in association with inflammatory cell infiltrates.IL-18 and IL-1 display more diffuse dermal staining. Elevated Ki-67 labeling indicates increased keratinocyte proliferation in lesional skin. Images are representative of lesional tissue; primary and secondary antibody details and imaging quantification methods are provided in Materials and Methods.

**Figure 3:**
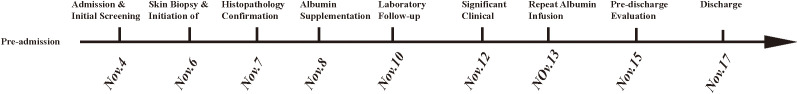
Clinical course and therapeutic timeline. The timeline illustrates the diagnostic and therapeutic progression from symptom onset to discharge. Key milestones include: Initial screening: RSV-PCR testing and initiation of IVIG (0.4 g/kg). Targeted therapy: Skin biopsy followed by the administration of oral acitretin (4 mg/day). Supportive care: Serial infusions of human albumin to correct hypoalbuminemia. Outcome: Clinical stabilization achieved by day 9, with discharge on hospital day 13.

Histopathology and treatment: Skin biopsy revealed subcorneal pustules and typical Kogoj pustules, accompanied by psoriasiform epidermal hyperplasia and papillary dermal edema (**Figures 1D–F**). Immunofluorescence analysis of lesional skin revealed marked expression of IL-36, IL-18, IL-1, and Ki-67, with IL-36 predominantly localized in the epidermis, particularly in pustular regions, and additional expression observed in the dermis associated with inflammatory infiltrates (**Figure 2**). On the second hospital day, the patient was treated with intravenous fluid replacement, intravenous immunoglobulin (IVIG, 0.4 g/kg), and albumin for supportive care. Oral acitretin was added on day 3 at a dose of 4 mg/day (approximately 0.3-0.5 mg/kg). Following treatment, body temperature normalized and the cutaneous lesions improved markedly, with no new pustule formation (**Figure 1G**). The patient was discharged in stable condition after a 13-day hospital stay (**Figures 1D, E**) and continued oral acitretin for approximately 2 weeks after discharge (**Figure 3**). In total, oral acitretin was administered for about 4 weeks, including 13 days during hospitalization. At the 1-month follow-up, the medication had been discontinued, and only faint residual erythema remained at the sites of previous lesions, with no evidence of pustular recurrence (**Figure 1H**).

## Discussion

From a mechanistic standpoint, respiratory syncytial virus (RSV)—a major etiologic agent of pediatric bronchopneumonia—elicits robust innate immune activation in respiratory epithelium via pattern-recognition receptors (e.g., TLRs, RIG-I-like receptors), triggering downstream NF-κB and inflammasome signaling (4, 6). This leads to the systemic release of proinflammatory cytokines, including IL-1β and IL-18. Notably, the IL-36 cytokine family is recognized as a central amplifier of neutrophilic inflammation in generalized pustular psoriasis (GPP) (5, 7). Importantly, such cytokine upregulation is also observed in GPP patients without infection, supporting that these findings are not specific to RSV. However, direct evidence linking RSV infection to the IL-36 pathway in human skin remains sparse, and the association is largely extrapolated from experimental models (8). Consequently, in susceptible hosts, viral infection may be best regarded as a systemic immunological stressor that lowers the inflammatory threshold, acting as a critical “second hit” rather than a definitively proven singular cause of GPP. Direct viral involvement of the skin was not assessed, which represents a limitation. In our patient, immunofluorescence of lesional skin showed marked expression of IL-36 and other IL-1 family cytokines, predominantly observed in regions enriched with inflammatory infiltrates (9). The predominant epidermal localization of IL-36 is consistent with its established role in keratinocyte-driven inflammation in GPP.

Infants possess both an immature epidermal barrier and a developing immune system, which may predispose them to infection-associated inflammatory amplification (10, 11). Although genetic testing for variants in IL36RN, CARD14, or related genes was not performed, IL36RN mutations remain a well-recognized cause of GPP, particularly in early-onset patients (5). Importantly, the prominent expression of IL-1 and IL-18 in lesional skin does not exclude the possibility of such underlying genetic susceptibility. The clinical presentation, marked by severe systemic inflammation and increased tissue expression of IL-1 and IL-18, suggests that a potent virus-triggered innate immune response may, in some young children, phenocopy a genetically determined GPP phenotype. This observation highlights that environmental triggers may suffice to drive a fulminant pustular flare even in the absence of confirmed monogenic mutations; however, this remains a hypothesis derived from a single case and requires validation in larger cohorts. In addition, circulating levels of IL-1, IL-18, and IL-36 were not measured in this patient; therefore, the contribution of systemic cytokine responses to the clinical manifestations cannot be fully determined.

Accurate discrimination between GPP and acute generalized exanthematous pustulosis (AGEP) carries direct therapeutic consequences (12). Here, the onset of pustules prior to cephalosporin exposure, the patient’s history of psoriasis, and the histopathological identification of Kogoj spongiform pustules collectively support a diagnosis of GPP over AGEP (1). The absence of eosinophils within the pustules and the predominance of neutrophils further support the diagnosis of GPP over AGEP. It should be acknowledged, however, that drug-related and infection-related pustuloses can exhibit clinical and histological overlap, and a contributory role of medication cannot be entirely ruled out.

Therapeutically, a combined regimen was employed. Oral acitretin targeted keratinocyte hyperproliferation, while intravenous immunoglobulin (IVIG) served to modulate the dysregulated immune response, potentially via Fc-receptor competition and neutralization of inflammatory mediators. Albumin supplementation corrected hypoalbuminemia, supporting volume homeostasis and barrier repair (2). Given the concurrent use of these interventions, the individual contribution of each component to clinical improvement cannot be ascertained from a single case.

Although anti-IL36 receptor therapy (e.g., spesolimab) has demonstrated efficacy in adults, its safety and dosing in infants remain undefined (13). After discussion with the family, biologic therapy was not pursued. The favorable outcome in this patient underscores that early recognition of a temporal viral trigger, confirmatory histopathology, and prompt immunomodulatory and supportive care can achieve rapid stabilization. Further studies are needed to clarify optimal management strategies and to elucidate the precise mechanistic links between viral infections and the IL-36 axis in pediatric GPP (14, 15).

## Data Availability

The original contributions presented in the study are included in the article/supplementary material. Further inquiries can be directed to the corresponding author.
